# Prevalence and factors associated with anxiety and depression among the older people in ethnic minority areas in southern China: a cross-sectional study

**DOI:** 10.3389/fpubh.2025.1494629

**Published:** 2025-06-24

**Authors:** Suyi Wu, Liu Yang, Haini Mo, Li Li, Zirong Li, Yanping Ying

**Affiliations:** The Department of Nursing, First Affiliated Hospital, Guangxi Medical University, Nanning, Guangxi, China

**Keywords:** older people, community, anxiety, depression, ethnic minority

## Abstract

**Background:**

Mental health issues among the older people are increasingly becoming a focus of societal concern, with depression and anxiety being common psychological problems that affect their quality of life and physical health. However, research on anxiety and depression among ethnic minorities in China remains relatively limited. This study investigates ethnic disparities in mental health among older adults (≥65 years) in China’s Guangxi Zhuang Autonomous Region, employing a Social Determinants of Health (SDH) framework.

**Methods:**

A cross-sectional study was conducted using a multi-stage stratified sampling method among 1,671 older individuals aged 65 and above in five communities in Guangxi from April to May 2024. A total of 1,550 completed questionnaires were collected. Descriptive analysis, univariate analysis, and two-factor logistic regression analysis were employed to explore the influencing factors of depression and anxiety among the older people in ethnic minority areas.

**Results:**

The prevalence rates of anxiety and depression were 11.42 and 15.94%, respectively. Logistic regression analysis indicated that being female, belonging to ethnic minorities (such as Jing, Yao, Mulao, and Zhuang), cognitive impairment, holding negative attitudes towards aging, and poor psychological resilience were common and significant predictors of both anxiety and depression. Support from children and access to medical insurance emerged as common protective factors against anxiety and depression.

**Conclusion:**

The incidence of anxiety and depression symptoms among the older people in the Guangxi Zhuang Autonomous Region is relatively high. Specifically, ethnic minorities such as Jing, Yao, Mulao, and Zhuang exhibited a higher likelihood of experiencing anxiety and depression compared to non-ethnic minorities like Han. This finding highlights the multiple mental health challenges faced by these groups in terms of socioeconomic status, culture, education, and healthcare access. The government should prioritize the mental health of ethnic minorities by optimizing the allocation of social resources and promoting culturally adapted mental health services to address these challenges.

## 1 Introduction

With globalization, population aging is accelerating at an unprecedented rate, turning healthy aging into a global challenge (1). In China, by 2023, the population aged 65 and above surpassed 217 million, representing 15.4% of the total population, with a dependency ratio of 22.5% (2). This marks China’s entry into a deep aging stage, with a higher-than-average global aging level. This demographic shift poses challenges to socio-economic development but also creates opportunities for public health system construction, particularly in addressing the health and social needs of the older people (3). Mental health issues among the older people, particularly depression and anxiety, which have higher incidence rates compared to other age groups, cannot be ignored (4). The latest China Mental Health Survey shows that the lifetime prevalence of anxiety disorders in China is 7.6%, the highest reported (5). In 2012, depression became the second largest disease burden in China, and globally, it accounted for 10.3% of the total disease burden, ranking first (6). In China, the overall prevalence of depression among the older people is 22.7% (7). These psychological problems not only threaten the physical and mental health of the older people but also increase the risk of disability and suicide, imposing a significant economic burden on individuals and society (8).

China is a unified multi-ethnic nation, with ethnic minorities inhabiting 60% of the country’s territory. Minority regions have a higher ageing rate than the national average. Take the Guangxi Zhuang Autonomous Region as an example. It has the largest population of ethnic minorities in China. The proportion of people aged 65 and above is 13.81% (9), but there is an uneven distribution of mental health resources in these minority regions. For example, the psychiatric resources in the western regions are only 1/7 to 1/11 of those in the eastern regions (10). Data from the 2021 China Mental Health Survey shows that the 12-month treatment rate for depressive disorders was only 9.5%, with less than 0.5% receiving adequate treatment (11). Such structural inequality may lead to a higher rate of untreated mental illnesses among older people ethnic minorities. In addition, older people from ethnic minority groups face significant barriers in accessing mental health services due to language differences, cultural practices, and social exclusion (12). Minority regions also face socioeconomic and status segregation. Studies show that in border minority areas, older people often have low education and income levels. Combined with poor transportation, these factors lead to poor accessibility to health services, reflecting health inequities that disproportionately affect the poor and less educated (13).

The health status of ethnic minority groups is pivotal not only to their individual health and quality of life but also to the preservation of amicable ethnic relations and the stability of the nation. However, among individuals from historically marginalized ethnic groups, depression and anxiety are often underreported and undertreated, with these conditions frequently presenting with greater severity. Although prevalence rates of anxiety and depression appear relatively similar across different ethnic groups, extant research suggests that individuals from minoritized racial and ethnic backgrounds tend to experience more persistent mental illness and have lower rates of treatment utilization (14). In addition, there is a paucity of research on the mental health of ethnic minorities within China, particularly in underdeveloped regions such as Guangxi, where mental health services remain comparatively underdeveloped relative to more socioeconomically advanced provinces. Against this backdrop, this study systematically explores for the first time the ethnic differences in mental health among older people from ethnic minorities under the framework of Social Determinants of Health (SDH), providing evidence support for the formulation of precise intervention strategies.

## 2 Method

### 2.1 Samples and procedure

**Figure 1:**
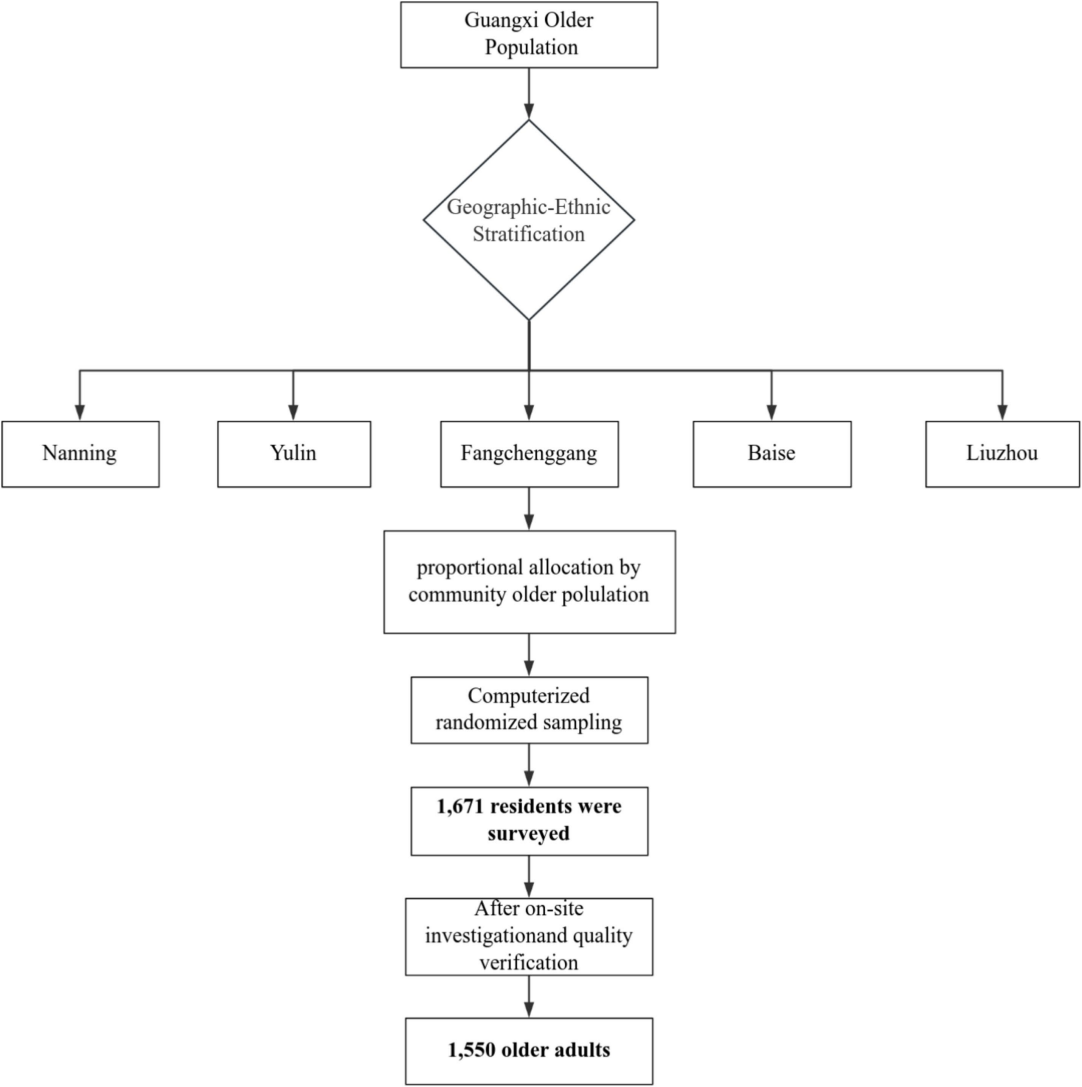
Flow chart of the sampling procedure in this study.

Data were collected between April and May 2024 in Guangxi Zhuang Autonomous Region, a southern Chinese border area characterized by ethnic diversity, including the Zhuang, Yao, Miao, Dong, Hui, Mulao, and Jing communities. Employing a multi-stage stratified sampling framework, we focused on five municipalities (Nanning, Yulin, Fangchenggang, Baise, Liuzhou) selected through geographic-ethnic stratification to represent predominant minority groups: Zhuang (Nanning), Han (Yulin), Jing (Fangchenggang), Yao (Baise), and Mulao (Liuzhou). Communities within each city were proportionally sampled based on older adult population registries. Using computer-generated random sampling, we identified potential participants aged ≥65 years who had resided locally for >6 months. Exclusion criteria included dementia or severe mental disorders, as reported by family members. The minimum sample size was determined using (15):$\;\text{Sample size}=\frac{{Z_{1-\alpha /2}}^{2}p(1-p)}{d^{2}}$, with *Z*_1-_α_/2_ = 1.96, α = 0.05 and *d* = 0.05, *p =* 0.211 (the prevalence of anxiety among the older adults was reported to be 21.1% based on previous studies) (16). Accounting for 20% non-response, we recruited 1,671 participants via telephone screening. After excluding 121 non-compliant cases, 1,550 subjects comprised the final sample. While stratification enhanced representativeness, the telephone recruitment may have excluded individuals with hearing difficulties or limited technology access. The sampling framework of this study as shown in Figure 1.

### 2.2 Variables and instruments

#### 2.2.1 Dependent variable

(1) Anxiety status was assessed using the Generalized Anxiety Disorder (GAD-7) questionnaire. This scale has strong reliability, validity, repeatability and applicability among different populations and has been widely used in clinical practice (17). A score ≥5 is regarded as the presence of an anxious state, with 5, 10 and 15 points, respectively, serving as the cut-off points for mild, moderate and severe anxiety (18). (2) Depressive status was evaluated using the Patient Health Questionnaire-9 Questionnaire. This questionnaire is simple in design, easy to administer and score, and widely adopted in community mental health screenings. A score of 5 or above indicates a positive screening result and may reflect depressive symptoms. Scores of 5–9, 10–14 and 15–27, respectively, indicate the possible existence of mild, moderate and severe depression (19).

#### 2.2.2 Independent variable

The Social Determinants of Health (SDH) encompass a wide range of factors that influence health outcomes, including economic, social, environmental, and psychosocial dimensions (20). These determinants are fundamental in shaping individuals’ health and disease trajectories, extending from birth through aging and encompassing all aspects of life, including growth, living conditions, and work environments (21). While SDH are primarily utilized to trace the origins of diseases, they also provide a comprehensive framework for understanding health disparities. For instance, a study previously employed SDH to examine the prevalence of depression among African American adults in the United States (22). In this study, SDH were categorized into five dimensions: individual characteristics, behavioral patterns, social support networks, socioeconomic status, and other social structural factors. Drawing on this framework, the current study identifies variables that may influence anxiety and depression among older adults in Guangxi: (1) individual characteristics: age, gender, ethnics, chronic diseases, cognitive impairment; (2) behavioral patterns: living alone or not, exercise frequency, psychological resilience, attitude towards aging; (3) social support networks: marital status, relationships with neighbors, support from couple, support from children, and support from friends; (4) socioeconomic status: residential area, employment, education level; (5) other social structural factors: medical insurance. Cognitive impairment was assessed using the Ascertain Dementia 8 (AD8). An AD8 score of ≥2 suggests cognitive impairment. The study found that the AD8 is particularly suitable for cognitive function screening in older community populations (23), and it is a quick, simple, sensitive, and reliable screening method (24).

#### 2.2.3 Statistical analysis

Data were analyzed using SPSS software version 26.0 for iOS, with a *p*-value < 0.05 considered statistically significant. Descriptive statistics were employed for summarization, including frequencies and percentages for categorical data, as well as means and standard deviations for continuous data. Chi-square tests were used for bivariate analysis to identify significant differences in categorical variables between groups. Statistically significant variables from univariate analysis were included in the logistic regression model. Two binary logistic regression models were established to analyze the association between depression, anxiety, and predictor variables, calculating unadjusted odds ratios (UOR), adjusted odds ratios (AOR) and their 95% confidence intervals (CI).

#### 2.2.4 Quality control

Subjects were selected, and questionnaires were collected according to established inclusion and exclusion criteria, ensuring on-site retrieval. All investigators underwent standardized training before the survey. During data collection, researchers were limited to clarifying and guiding doubts, strictly prohibiting any prompting, or influencing of respondents’ answers. To ensure accuracy, two research assistants were assigned to data entry. In case of discrepancies, a third party would review the original records and make corrections.

## 3 Results

### 3.1 Sample characteristics

Altogether, we invited 1,671 older people to participate in the study and 1,550 agreed and completed the survey questionnaire (response rate: 92.76%). The study sample mainly comprised older adults aged 65–74 years (61.0%), with an average age of 73.77 years (SD = 6.47), and the oldest respondent was 101 years old. Regarding gender distribution, 57.4% were female and 42.6% were male. Most participants were married (67.7%) and had an elementary school level (54.4%). In this study, the Han ethnic group, as a non-minority ethnic group, has the largest population, accounting for 26.8%. Meanwhile, the proportions of ethnic minorities such as the Jing, Yao, Mulao and Zhuang were 15.7, 17.9, 13.2 and 26.5%, respectively. Characteristics of the survey sample and prevalence rates of anxiety and depression among different subgroups are shown in Table 1. Chi-square tests revealed statistically significant differences (*P*_1,2_<0.05) in anxiety and depression symptoms across factors such as age, gender, ethnics, chronic diseases, cognitive impairment, exercise frequency, psychological resilience, aging attitude, marital status, relationship with neighbors, support from couple, Support from children, education level and medical insurance. Besides, Chi-square test results also indicated statistically significant differences (*P*_1_ = 0.035) in anxiety among older people based on the residential area.

**Table 1:** Descriptive analysis of the general characteristics of the older people in ethnic minority areas by anxiety and depression.

Variables	Total	Anxiety			Depression		
	*N* (%)	No *n* (%)	Yes *n* (%)	*P*_1_	No *n* (%)	Yes *n* (%)	*P*_2_
Observations	1,550	1,373	177		1,303	247	
Age							
65 ~ 74	945 (61.0)	847 (61.7)	98 (55.4)	0.018	810 (62.2)	135 (54.7)	0.004
75 ~ 84	491 (31.7)	434 (31.6)	57 (32.2)		409 (31.4)	82 (33.2)	
≥85	114 (7.4)	92 (6.7)	22 (12.4)		84 (6.4)	30 (12.1)	
Gender							
Male	660 (42.6)	609 (44.4)	51 (28.8)	<0.001	592 (45.4)	68 (27.5)	<0.001
Female	890 (57.4)	764 (55.6)	126 (71.2)		711 (54.6)	179 (72.5)	
Ethnics							
Han	415 (26.8)	397 (28.9)	18 (10.2)	<0.001	389 (29.9)	26 (10.5)	<0.001
Jing	244 (15.7)	201 (14.6)	43 (24.3)		191 (14.7)	53 (21.5)	
Yao	277 (17.9)	248 (18.1)	29 (16.4)		237 (1/8.2)	40 (16.2)	
Mulao	204 (13.2)	182 (13.3)	22 (12.4)		165 (12.7)	39 (15.8)	
Zhuang	410 (26.5)	345 (25.1)	65 (36.7)		321 (24.6)	89 (36.0)	
Chronic disease							
None	412 (26.6)	383 (27.9)	29 (16.4)	0.001	376 (28.9)	36 (14.6)	<0.001
One or two	954 (61.5)	837 (61.0)	117 (66.1)		792 (60.8)	162 (65.6)	
Three or more	184 (11.9)	153 (11.1)	31 (17.5)		135 (10.4)	49 (19.8)	
Cognitive impairment							
Absent	1,019 (65.7)	955 (69.6)	64 (36.2)	<0.001	929 (71.3)	90 (36.4)	<0.001
Present	531 (34.3)	418 (30.4)	113 (63.8)		374 (28.7)	157 (63.6)	
Living alone							
No	1,362 (87.9)	1,209 (88.1)	153 (86.4)	0.536	1,153 (88.5)	209 (84.6)	0.087
Yes	188 (12.1)	164 (11.9)	24 (13.6)		150 (11.5)	38 (15.4)	
Exercise frequency							
Never	764 (49.3)	648 (47.2)	116 (65.5)	<0.001	605 (46.4)	159 (64.4)	<0.001
Occasionally	495 (31.9)	455 (33.1)	40 (22.6)		443 (34.0)	52 (21.1)	
Regularly	291 (18.8)	270 (19.7)	21 (11.9)		255 (19.6)	36 (14.6)	
Psychological resilience							
Good	712 (45.9)	666 (48.5)	46 (26.0)	<0.001	655 (50.3)	57 (23.1)	<0.001
Moderate	496 (32.0)	447 (32.6)	49 (27.7)		413 (31.7)	83 (33.6)	
Poor	342 (22.1)	260 (18.9)	82 (46.3)		235 (18.0)	107 (43.3)	
Aging attitude							
Positive	745 (48.1)	691 (50.3)	54 (30.5)	<0.001	661 (50.7)	84 (34.0)	<0.001
Moderate	450 (29.0)	388 (28.3)	62 (35.0)		365 (28.0)	85 (34.4)	
Negative	355 (22.9)	294 (21.4)	61 (34.5)		277 (21.3)	78 (31.6)	
Marital status							
Married	1,049 (67.7)	946 (68.9)	103 (58.2)	0.004	912 (70.0)	137 (55.5)	<0.001
Single	501 (32.3)	427 (31.1)	74 (41.8)		391 (30.0)	110 (44.5)	
Relationship with neighbors							
Poor	344 (22.2)	286 (20.8)	58 (32.8)	<0.001	268 (20.6)	76 (30.8)	<0.001
Well	1,206 (77.8)	1,087 (79.2)	119 (67.2)		1,035 (79.4)	171 (69.2)	
Support from couple							
Absent	533 (34.4)	454 (33.1)	79 (44.6)	0.002	416 (31.9)	117 (47.4)	<0.001
Present	1,017 (65.6)	919 (66.9)	98 (55.4)		887 (68.1)	130 (52.6)	
Support from children							
Absent	68 (4.4)	50 (3.6)	18 (10.2)	<0.001	49 (3.8)	19 (7.7)	0.006
Present	1,482 (95.6)	1,323 (96.4)	159 (89.8)		1,254 (96.2)	228 (92.3)	
Support from friends							
Absent	297 (19.2)	260 (18.9)	37 (20.9)	0.531	245 (18.8)	52 (21.1)	0.41
Present	1,253 (80.8)	1,113 (81.1)	140 (79.1)		1,058 (81.2)	195 (78.9)	
Education level							
Junior high school and above	89 (5.7)	85 (6.2)	4 (2.3)	0.001	83 (6.4)	6 (2.4)	<0.001
Beginner level	302 (19.5)	273 (19.9)	29 (16.4)		274 (21.0)	28 (11.3)	
Elementary school level	843 (54.4)	753 (54.8)	90 (50.8)		711 (54.6)	132 (53.4)	
No formal or primary education	316 (20.4)	262 (19.1)	54 (30.5)		235 (18.0)	81 (32.8)	
Residential area							
Urban	521 (33.6)	449 (32.7)	72 (40.7)	0.035	428 (32.8)	93 (37.7)	0.143
Rural	1,029 (66.4)	924 (67.3)	105 (59.3)		875 (67.2)	154 (62.3)	
Employment							
Unemployed	499 (32.2)	452 (32.9)	47 (26.6)	0.088	426 (32.7)	73 (29.6)	0.333
Retired and having income	1,051 (67.8)	921 (67.1)	130 (73.4)		877 (67.3)	174 (70.4)	
Medical insurance							
Absent	411 (26.5)	315 (22.9)	96 (54.2)	<0.001	292 (22.4)	119 (48.2)	<0.001
Present	1,139 (73.5)	1,058 (77.1)	81 (45.8)		1,011 (77.6)	128 (51.8)	

P_1_ for the differences in prevalence of anxiety among participants with different characteristics; P_2_ for the differences in prevalence of depression among participants with different characteristics.

### 3.2 Distribution of anxiety and depression symptoms

**Figure 2:**
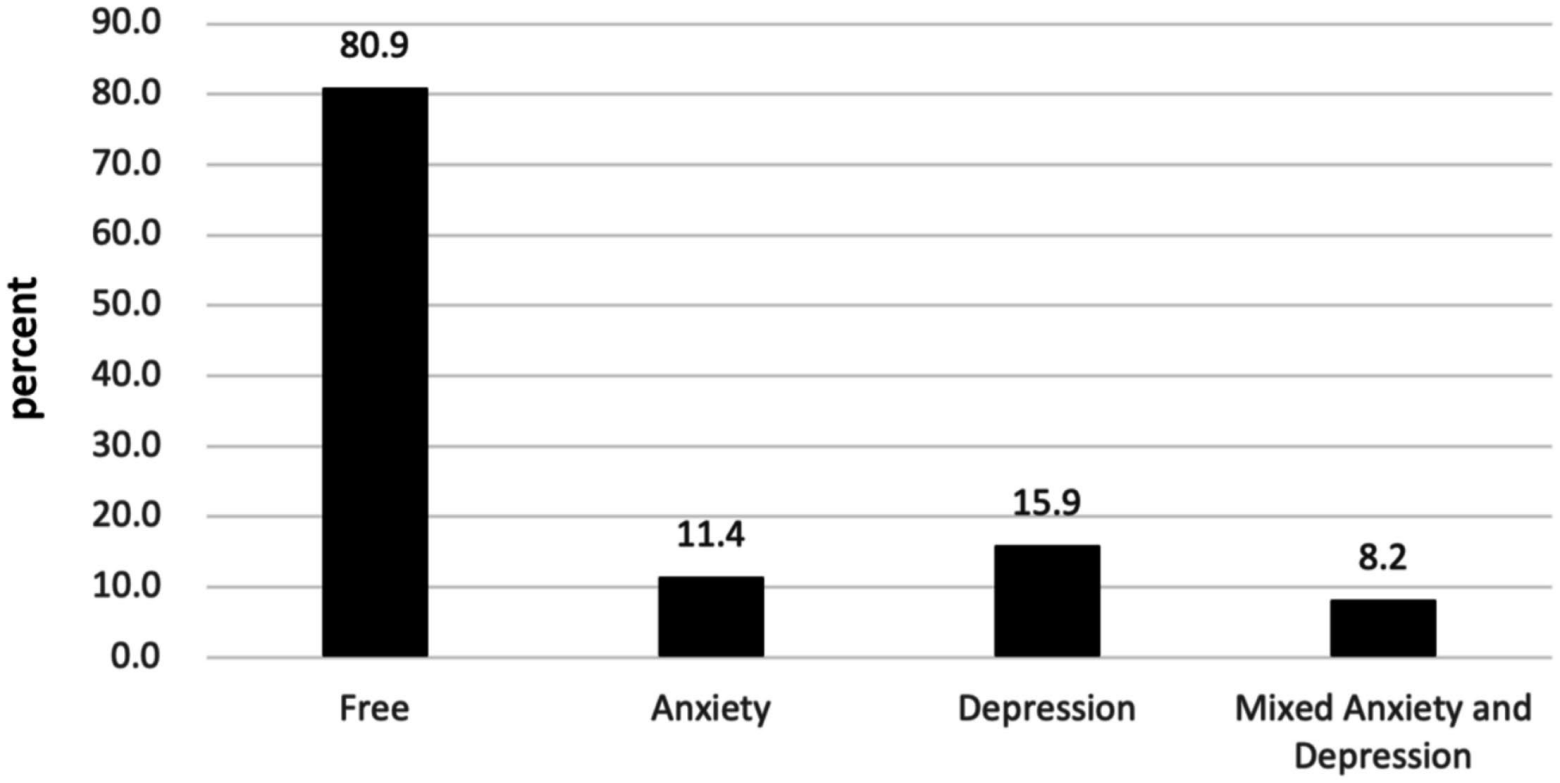
Psychological health problems among the studied group (*N* = 1,550). ‘Free,’ for group without anxiety or depression; ‘Anxiety’ for group with only anxiety; ‘Depression’ for group with only depression; and ‘Mixed Anxiety and Depression’ for group experiencing both conditions.

**Figure 3:**
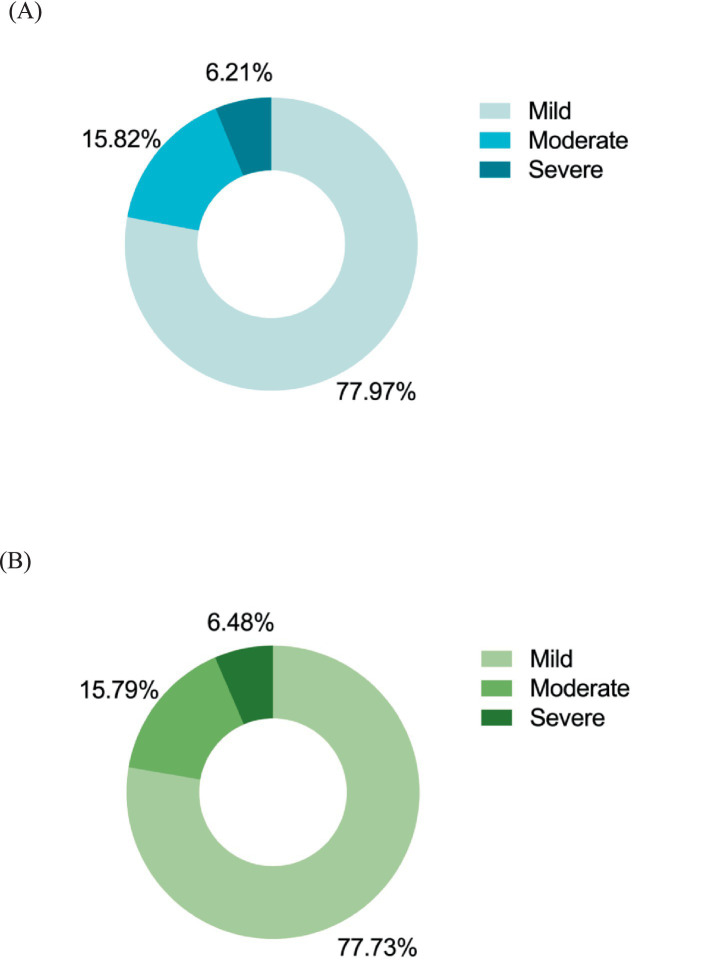
The prevalence of anxiety and depression of different severity. Mild, with GAD and PHQ scores ranging from 5 to 9; Moderate, GAD and PHQ scores in the 10–14 range; Sever, GAD and PHQ scores of 15 and above. **(A)** The rate of various severity levels of anxious older people (*N* = 177). **(B)** The rate of various severity levels of depressed older people (*N* = 247).

**Figure 4:**
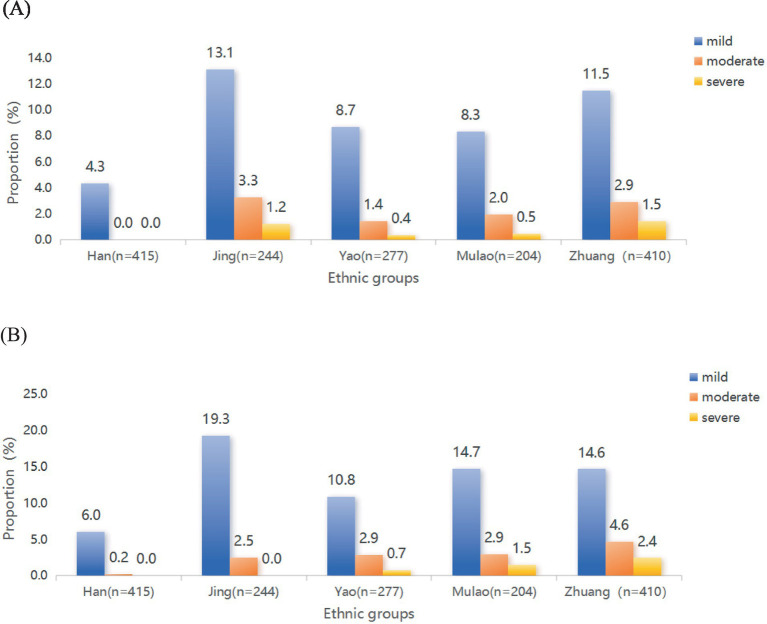
The prevalence of varying degrees of anxiety and depression among the older people in ethnic minority areas. **(A)** The rate of different severities of anxiety among multiple ethnic groups. **(B)** The rate of different severities of depression among multiple ethnic groups.

Figures 2, 3 illustrates the mental health characteristics of the participants. Comprehensive analysis shows that 11.42% of participants exhibited anxiety symptoms, 15.94% exhibited depression symptoms, and 8.19% reported mixed anxiety and depressive disorder. Among the older adult population with anxiety and depression, the proportion of mild cases is the largest, accounting for 77.97 and 77.73%, respectively. Figure 4 displays the distribution of anxiety and depression symptoms among different ethnic groups. The Jing nationality shows the highest incidence of mild to moderate symptoms and the total incidence rate, while the Zhuang ethnic group has the highest incidence of severe anxiety. For depression, The Jing nationality has the highest rate of mild depression, while the Zhuang nationality has the highest rates for moderate and severe depression. Collectively, ethnic minority groups demonstrated significantly higher overall prevalence rates of anxiety and depression compared to the Han Chinese population. This disparity persisted across all severity levels, with minority populations exhibiting elevated incidence rates for mild, moderate, and severe symptom presentations.

### 3.3 Correlates of anxiety and depression symptoms

Logistic regression analyses were conducted to test for significant predictors of anxiety, as detailed in Table 2. Based on the results of the bivariate analysis, significant variables were accessed in the regression model. In the multivariable model, those of female gender showed a higher risk factor for anxiety (AOR: 1.836, 95% CI: 1.213–2.78). The older adults from the Jing (AOR: 3.199, 95%CI: 1.629–6.284), Yao (AOR: 2.304, 95%CI: 1.15–4.619), Mulao (AOR: 1.999, 95% CI: 1.113–4.078) and Zhuang ethnic groups (AOR: 2.373, 95% CI: 1.305–4.316) all exhibit a heightened vulnerability to anxiety than those older adults from Han ethnic. Furthermore, those with cognitive impairment (AOR: 2.621, 95%CI: 1.804 ~ 3.809), poor psychological resilience (AOR: 2.276, 95%CI: 1.464 ~ 3.544) and a negative attitude towards aging (AOR: 1.555, 95%CI: 1.213 ~ 2.455) were also at an increased risk for anxiety. We also found that older residents receiving support from their children (AOR: 0.489, 95%CI: 0.308 ~ 0.778), living in rural (AOR: 0.434, 95%CI: 0.217 ~ 0.87) and having health insurance (AOR: 0.424, 95%CI: 0.287 ~ 0.626) exhibited a reduced likelihood of anxiety.

**Table 2:** Simple and multiple logistic regression for relationships between related variables and anxiety of the older people in ethnic minority areas (*N* = 1,550).

Variables	Categories	UOR (95% CI)	AOR (95% CI)	*P*-value
Age	65 ~ 74	1.0	1.0	
	75 ~ 84	1.135(0.803 ~ 1.605)	0.912(0.612 ~ 1.359)	0.652
	≥85	2.067(1.241 ~ 3.442)	1.385(0.756 ~ 2.538)	0.291
Gender	Male	1.0	1.0	
	Female	1.969(1.399 ~ 2.773)	1.836(1.213 ~ 2.78)	0.004**
Ethnics	Han	1.0	1.0	
	Jing	4.718(2.653 ~ 8.392)	3.199(1.629 ~ 6.284)	0.001**
	Yao	2.579(1.403 ~ 4.743)	2.304(1.15 ~ 4.619)	0.019*
	Mulao	2.666(1.396 ~ 5.092)	1.999(1.113 ~ 4.078)	0.047*
	Zhuang	4.155(2.418 ~ 7.142)	2.373(1.305 ~ 4.316)	0.005**
Chronic disease	None	1.0	1.0	
	One or two	1.846(1.208 ~ 2.821)	1.186(0.735 ~ 1.912)	0.485
	Three or more	2.676(1.559 ~ 4.592)	1.418(0.749 ~ 2.684)	0.284
Cognitive impairment	Absent	1.0	1.0	
	Present	4.034(2.907 ~ 5.597)	2.621(1.804 ~ 3.809)	<0.001***
Exercise frequency	Regularly	1.0	1.0	
	Occasionally	1.13(0.653 ~ 1.958)	0.82(0.45 ~ 1.492)	0.516
	Never	2.302(1.416 ~ 3.742)	1.173(0.659 ~ 2.087)	0.588
Psychological resilience	Good	1.0	1.0	
	Moderate	1.587(1.043 ~ 2.415)	0.685(0.42 ~ 1.117)	0.13
	Poor	4.566(3.096 ~ 6.734)	2.276(1.464 ~ 3.54)	<0.001***
Aging attitude	Positive	1.0	1.0	
	Moderate	2.045(1.391 ~ 3.006)	1.296(0.833 ~ 2.017)	0.25
	Negative	2.655(1.796 ~ 3.925)	1.555(1.213 ~ 2.455)	0.038*
Marital status	Married	1.0	1.0	
	Single	1.592(1.156 ~ 2.191)	1.785(0.989 ~ 3.22)	0.054
Relationship with neighbors	Poor	1.0	1.0	
	Well	0.54(0.384 ~ 0.758)	0.857(0.567 ~ 1.294)	0.463
Support from couple	Absent	1.0	1.0	
	Present	0.554(0.404 ~ 0.759)	1.564(0.854 ~ 2.864)	0.147
Support from children	Absent	1.0	1.0	
	Present	0.358(0.25 ~ 0.511)	0.489(0.308 ~ 0.778)	0.003**
Education level	Junior high school and above	1.0	1.0	
	Beginner level	2.257(0.772 ~ 6.603)	1.903(0.623 ~ 5.812)	0.259
	Elementary school level	2.54(0.91 ~ 7.088)	1.567(0.529 ~ 4.64)	0.418
	No formal or primary education	4.38(1.541 ~ 12.449)	1.227(0.388 ~ 3.873)	0.728
Residential area	Urban	1.0	1.0	
	Rural	0.709(0.514 ~ 0.976)	0.434(0.217 ~ 0.87)	0.019*
Medical insurance	Absent	1.0	1.0	
	Present	0.251(0.182 ~ 0.346)	0.424(0.287 ~ 0.626)	<0.001***

UOR, unadjusted odds ratio; AOR, adjusted odds ratio (adjusted for all variables); *P-value < 0.05, **P-value < 0.01, ***P-value < 0.001.

In the depression group, multiple logistic regression analysis identified nine significant factors associated with depressive symptoms, as detailed in Table 3. The findings indicate that older women (AOR: 1.632, 95% CI: 1.133 ~ 2.349) are at a higher risk of developing depression compared to men. Additionally, certain ethnic groups, including the Jing (AOR: 2.222, 95% CI: 1.226 ~ 4.026), Yao (AOR: 2.498, 95% CI: 1.369 ~ 4.559), Mulao (AOR: 3.195, 95% CI: 1.751 ~ 5.83), and Zhuang (AOR: 2.327, 95% CI: 1.39 ~ 3.894), were identified as having a higher likelihood of experiencing depressive symptoms. The presence of chronic diseases (AOR_1_: 1.597, 95% CI_1_: 1.038 ~ 2.456; AOR_2_:2.442, 95% CI_2_: 1.394 ~ 4.278) and cognitive impairment (AOR: 2.94, 95% CI: 2.122 ~ 4.073) were also found to be significantly associated with an increased risk of depression. Psychological resilience emerged as a crucial factor, with lower levels of resilience (AOR: 2.839, 95% CI: 1.903 ~ 4.236) and negative attitude (AOR: 1.455, 95% CI: 1.171 ~ 2.18) of aging identified as strong predictors of depression. Conversely, occasional physical exercise (AOR: 0.545, 95% CI: 0.357 ~ 0.833), support from children (AOR: 0.619, 95% CI: 0.405 ~ 0.945), and access to medical insurance (AOR: 0.583, 95% CI: 0.411 ~ 0.828) were found to be protective factors against depression.

**Table 3:** Simple and multiple logistic regression for relationships between related variables and depression of the older people in ethnic minority areas (*N* = 1,550).

Variables	Categories	UOR (95% CI)	AOR (95% CI)	*P*-value
Age	65 ~ 74	1.0	1.0	
	75 ~ 84	1.203(0.892 ~ 1.622)	0.903(0.638 ~ 1.278)	0.565
	≥85	2.143(1.36 ~ 3.377)	1.076(0.623 ~ 1.859)	0.792
Gender	Male	1.0	1.0	
	Female	2.192(1.624 ~ 2.958)	1.632(1.133 ~ 2.349)	0.008**
Ethnics	Han	1.0	1.0	
	Jing	4.152(2.518 ~ 6.846)	2.222(1.226 ~ 4.026)	0.008**
	Yao	2.525(1.502 ~ 4.245)	2.498(1.369 ~ 4.559)	0.003**
	Mulao	3.536(2.084 ~ 6)	3.195(1.751 ~ 5.83)	<0.001***
	Zhuang	4.148(2.615 ~ 6.579)	2.327(1.39 ~ 3.894)	0.001**
Chronic disease	None	1.0	1.0	
	One or two	2.136(1.459 ~ 3.128)	1.597(1.038 ~ 2.456)	0.033*
	Three or more	3.791(2.362 ~ 6.084)	2.442(1.394 ~ 4.278)	0.002**
Cognitive impairment	Absent	1.0	1.0	
	Present	4.333(3.257 ~ 5.765)	2.94(2.122 ~ 4.073)	<0.001***
Exercise frequency	Never	1.0	1.0	
	Occasionally	0.447(0.319 ~ 0.625)	0.545(0.357 ~ 0.833)	0.005**
	Regularly	0.537(0.364 ~ 0.794)	1.04(0.647 ~ 1.673)	0.87
Psychological resilience	Good	1.0	1.0	
	Moderate	2.309(1.613 ~ 3.307)	1.129(0.743 ~ 1.716)	0.57
	Poor	5.232(3.671 ~ 7.457)	2.839(1.903 ~ 4.236)	<0.001***
Aging attitude	Positive	1.0	1.0	
	Moderate	1.833(1.321 ~ 2.543)	1.064(0.725 ~ 1.561)	0.753
	Negative	2.216(1.579 ~ 3.109)	1.455(1.171 ~ 2.18)	0.039*
Marital status	Married	1.0	1.0	
	Single	1.873(1.419 ~ 2.472)	1.547(0.926 ~ 2.582)	0.095
Relationship with neighbors	Poor	1.0	1.0	
	Well	0.583(0.431 ~ 0.788)	0.889(0.614 ~ 1.288)	0.534
Support from couple	Absent	1.0	1.0	
	Present	0.446(0.338 ~ 0.589)	1.012(0.599 ~ 1.709)	0.965
Support from children	Absent	1.0	1.0	
	Present	0.398(0.288 ~ 0.55)	0.619(0.405 ~ 0.945)	0.026*
Education level	Junior high school and above	1.0	1.0	
	Beginner level	1.414(0.566 ~ 3.53)	1.066(0.405 ~ 2.804)	0.897
	Elementary school level	2.568(1.099 ~ 6.004)	1.691(0.68 ~ 4.208)	0.259
	No formal or primary education	4.768(2.005 ~ 11.338)	1.562(0.593 ~ 4.115)	0.367
Medical insurance	Absent	1.0	1.0	
	Present	0.311(0.234 ~ 0.412)	0.583(0.411 ~ 0.828)	0.003**

UOR, unadjusted odds ratio; AOR, adjusted odds ratio (adjusted for all variables); *P-value < 0.05, **P-value < 0.01, ***P-value < 0.001.

## 4 Discussion

The prevalence of anxiety symptoms among the older adults in ethnic minority regions of Guangxi, China is 11.42%, and the prevalence of depressive symptoms is 15.94%. The observed higher prevalence of depression in this study compared to urban Sri Lankan older adults (13.9%) may be due to the age distribution (25). The study’s depression rate is lower than in other developing countries such as India (52.5%) (26), Nepal (57.8%) (27), rural Egypt (44.4%) (28), and Ethiopia (45%) (29), possibly due to the socioeconomic status of the study populations (30). A study in Botswana reported a higher anxiety prevalence of 18.5%, which may be related to a higher proportion of older adults with chronic diseases and social impairments, affecting their ability to access quality healthcare (31). Despite lower detection rates of anxiety and depression compared to other developing countries, the rates in Guangxi Zhuang Autonomous Region remain significant, underscoring the necessity for research and attention to the mental health of the older people in ethnic minority areas of China.

This study identifies elevated depression and anxiety prevalence among Guangxi’s ethnic minorities (Jing, Zhuang, Mulao, Yao) compared to the Han majority (Tables 2, 3), aligning with Western China’s mental health disparity patterns (32). The Zhuang ethnic group is the largest ethnic minority group in Guangxi. As a distinct ethnic group with their own language, cultural and linguistic differences may present communication and adaptation challenges when interacting with the broader society (33). For the Jing ethnic group, we speculated that their proximity to Vietnam and the resulting environmental, cultural, and customary similarities may have an impact on the mental health of the older adults (34). The majority of older individuals from the Mulao and Yao ethnic groups reside in remote mountainous areas, which are characterized by poor transportation and limited access to information. These geographical and infrastructural limitations significantly impede their ability to access adequate medical security and mental health services (35). Structural socioeconomic inequalities exhibit robust associations with mental health disparities, particularly in economically underdeveloped multiethnic regions like Guangxi (30, 36). These vulnerabilities are exacerbated by persistent social exclusion and identity struggles, as evidenced by Snowden and Hannah’s frameworks on minority mental health (37, 38). Despite regional development initiatives, the interplay of cultural marginalization and socioeconomic deprivation perpetuates psychological distress among ethnic elders, necessitating interventions that address both biomedical service gaps and sociocultural determinants (Table 4).

**Table 4:** Descriptive analysis of the general characteristics of the older people in different ethnic groups (*N* = 1,550).

Variables	Total	Ethnic groups [*n*(%)]				
		Han	Jing	Yao	Mulao	Zhuang
Observations	1,550	415	244	277	204	410
Gender						
Male	660 (42.6)	193(46.51)	116(47.54)	98(35.38)	87(42.65)	166(40.49)
Female	890 (57.4)	222(53.49)	128(52.46)	179(64.62)	117(57.35)	244(59.51)
Chronic disease						
None	412 (26.6)	171(41.20)	29(11.89)	40(14.44)	69(33.82)	103(25.12)
One or two	954 (61.5)	227(54.70)	149(61.07)	177(63.90)	125(61.27)	276(67.32)
Three or more	184 (11.9)	17(4.10)	66(27.05)	60(21.66)	10(4.90)	31(7.56)
Cognitive impairment						
Absent	1,019 (65.7)	323(77.83)	112(45.90)	192(69.31)	154(75.49)	238(58.05)
Present	531 (34.3)	92(22.17)	132(54.10)	85(30.69)	50(24.51)	172(41.95)
Exercise frequency						
Never	764 (49.3)	198(47.71)	132(54.10)	95(34.30)	82(40.20)	257(62.68)
Occasionally	495 (31.9)	127(30.60)	80(32.79)	103(37.18)	98(48.04)	87(21.22)
Regularly	291 (18.8)	90(21.69)	32(13.11)	79(28.52)	24(11.76)	66(16.00)
Psychological resilience						
Good	712 (45.9)	245(59.04)	60(24.59)	156(56.32)	69(33.82)	182(44.39)
Moderate	496 (32.0)	97(23.37)	138(56.56)	79(28.52)	77(37.75)	105(25.61)
Poor	342 (22.1)	73(17.59)	46(18.85)	42(15.16)	58(28.43)	123(30.00)
Aging attitude						
Positive	745 (48.1)	212(51.08)	62(25.41)	169(61.01)	117(57.35)	185(45.12)
Moderate	450 (29.0)	92(22.17)	110(45.08)	77(27.80)	51(25.00)	120(29.27)
Negative	355 (22.9)	111(26.75)	72(29.51)	31(11.19)	36(17.65)	105(25.61)
Support from children						
Absent	68 (4.4)	37(8.92)	16(6.56)	32(11.55)	56(27.45)	94(22.93)
Present	1,482 (95.6)	378(91.08)	228(93.44)	245(88.45)	148(72.55)	316(77.07)
Residential area						
Urban	521 (33.6)	54(13.01)	175(71.72)	218(78.70)	35(17.16)	39(9.51)
Rural	1,029 (66.4)	361(86.99)	69(28.28)	59(21.30)	169(82.84)	371(90.49)
Medical insurance						
Absent	411 (26.5)	65(15.66)	77(31.56)	51(18.41)	92(45.10)	126(30.73)
Present	1,139 (73.5)	350(84.34)	167(68.44)	226(81.59)	112(54.90)	284(69.27)
PHQ-9 score						
PHQ-9 < 5	1,303(84.06)	389(93.73)	191(78.28)	237(85.56)	165(80.88)	321(78.29)
PHQ-9 ≥ 5	247(15.94)	26(6.27)	53(21.72)	40(14.44)	39(19.12)	89(21.71)
GAD-7 score						
GAD-7 < 5	1,373(88.58)	397(95.66)	201(82.38)	248(89.53)	182(89.22)	345(84.15)
GAD-7 ≥ 5	177(11.42)	18(4.34)	43(17.62)	29(10.47)	22(10.78)	65(15.85)

Chronic illnesses significantly elevate depression risk in older adults, particularly among ethnic minorities. Meta-analytic evidence indicates that poor self-rated health and cardiovascular multimorbidity substantially increase depression susceptibility (39). Similarly, older adults with elevated scores on the AD8 scale also exhibit an increased susceptibility to anxiety and depressive disorders. Physical deterioration from chronic diseases often restricts daily functioning and social engagement, exacerbating mental health decline (40). This study highlights striking multimorbidity disparities, with Jing (88.12%) and Yao (85.56%) ethnic groups demonstrating exceptionally high rates. Contributory factors include culturally entrenched practices: sustained high-sodium/high-fat diets and normalized smoking/alcohol use as social rituals (41). These risk behaviors, compounded by systemic healthcare deficits – inadequate chronic disease screening and fragmented follow-up care in minority regions – create synergistic health threats (42). To disrupt this cycle, we propose: (1) culturally-tailored interventions integrating bilingual nutrition education (e.g., Zhuang language materials) into ethnic festivals to promote dietary modification and substance use reduction; (2) healthcare system strengthening through portable diagnostic deployment and village health worker training in early detection protocols; (3) equity-driven policy reforms prioritizing targeted subsidies and chronic care metrics in regional governance. Community-based chronic disease management coupled with mental health monitoring could optimize public health outcomes in aging populations (43, 44).

In this study, older individuals who with negative views of aging and poorer psychological resilience tend to be at a higher risk for anxiety and depressive symptoms, consistent with previous research (45, 46). Based on stereotype theory, Levy (47) proposed that aging attitudes influence the health status of the older people through physiological, behavioral, and psychological pathways. Internalized ageist stereotypes diminish self-efficacy and health behavior motivation (48–50), while multimorbidity exacerbates negative aging cognition through impaired self-care capacity (47, 51). Psychological resilience moderates this relationship, with enhanced emotional regulation buffering mental health impacts (52, 53). Our findings reveal critical disparities: Zhuang older adults demonstrated the poorer psychological resilience and more negative aging attitudes (Figure 4), correlating with their heightened anxiety/depression prevalence. To counteract these effects, we advocate: (1) An Intangible Cultural Heritage (ICH)-integrated intervention model utilizing traditional practices (e.g., ethnic dance, paper-cutting) to simultaneously preserve cultural identity and bolster self-worth; (2) Lifelong learning initiatives combining ethnic cultural studies with health literacy curricula to enable proactive aging engagement. These culturally grounded strategies target stereotype restructuring while promoting psychosocial resource accumulation, potentially disrupting the adverse aging perception-mental morbidity cycle.

This study corroborates the multifactorial protective effects against geriatric depression and anxiety. Regular physical exercise, particularly moderate-intensity aerobic and strength training, demonstrates dual benefits by enhancing physiological resilience and self-efficacy (54–57). Concurrently, filial support rooted in China’s cultural norms significantly mitigates mental health risks, with children serving as pivotal anchors in elders’ psychosocial support networks—a critical buffer against loneliness among ethnic minority populations (58–60). Notably, rural elders exhibited lower anxiety prevalence than urban counterparts (49), potentially attributable to traditional value systems that foster adaptive coping with life transitions (61, 62). These findings collectively underscore the necessity of culturally informed interventions that synergize biopsychosocial mechanisms to optimize mental health outcomes in aging populations.

Our article also illustrates that participation in medical insurance programs has a beneficial impact on mitigating symptoms of depression and anxiety. With insured middle-aged and older adults achieved higher life satisfaction compared to their uninsured counterparts (63, 64). Liu’s analysis confirms insurance’s critical role in geriatric mental health (65), while Baicker’s research highlights Medicaid expansion decreasing undiagnosed and untreated depression by 50 and 60%, respectively (66). However, Cheruvu and Chiyaka (67) observed elevated depression risks among insured seniors facing substantial out-of-pocket costs, suggesting coverage adequacy, not mere availability, determines mental health outcomes. These divergent findings likely stem from methodological variations, particularly in assessing financial toxicity thresholds. Our findings extend this discourse through ethnic disparity analysis (Figure 4): Han Chinese show highest insurance coverage (84.34%) versus Mulao minority’s lowest rates (54.90%), attributable to geographical remoteness compounding systemic barriers inadequate insurance promotion, complex reimbursement protocols, and income constraints limiting commercial insurance access. Crucially, basic insurance schemes in minority regions inadequately cover mental health services (68), maintaining suboptimal reimbursement rates for depression/anxiety treatments.

Furthermore, our findings demonstrate a significant association between female gender and the prevalence of anxiety and depressive symptoms, consistent with systematic reviews identifying gender as a critical predictor of mental health disparities among older adults (69). This gender disparity may be mediated by multifaceted determinants including socioeconomic status disparities, traumatic life experiences, and hormonal fluctuations (70–72). Community-based mental health initiatives should prioritize geriatric females through targeted interventions: implementing mental health literacy programs to enhance disease awareness, and establishing dedicated counseling channels (hotlines or in-person sessions) to provide timely psychological support and counseling.

This study identified the influencing factors of anxiety and depression among older adult from ethnic minorities and made relevant analyses on ethnic differences. Guided by Scott’s theory of weak ethnic groups (73), we proposes a culturally adaptive intervention framework addressing structural mental health disparities among ethnic minorities. The multipronged approach integrates: (1) a multilingual digital platform featuring AI-driven speech interaction (dialect recognition accuracy≥92%) and a cross-linguistic terminology repository spanning eight ethnic languages (Zhuang, Yao, Jing, etc.), enabling culturally congruent mental health screening; (2) a tiered training system for bilingual cultural coordinators (1.5 per 10,000 residents) requiring dual certification in ethnic linguistic proficiency and traditional healing practices, supported by mobile service kits and health behavior incentive programs; (3) a three-tier clinical network (village-town-city) incorporating digital PHQ-9/GAD-7 screening, VR-based relaxation therapy, and cross-cultural CBT, enhanced by blockchain medical records and UAV-delivered emergency supplies (≤12 h response); (4) policy innovations including formulating relevant laws to stipulate an 85% screening insurance coverage rate, as well as conducting cross-border intervention exchanges through the ASEAN Mental Health Alliance. This framework systematically bridges cultural competence gaps while addressing healthcare access inequities through technological, human resource, and policy synergies.

While this study provides novel insights into ethnic mental health disparities among older adults in China, several limitations warrant consideration. First, although validated scales were employed, cultural variations in symptom expression among ethnic minorities might affect measurement accuracy, as standardized cutoff scores may not fully account for culturally specific manifestations of distress. Second, the cross-sectional design precludes causal inferences regarding observed associations between social determinants and mental health outcomes. Third, while the SDH framework was comprehensively applied, unmeasured confounders such as historical trauma or acculturative stress—known mediators of minority mental health—were not assessed. These limitations underscore the need for longitudinal designs integrating mixed methods to elucidate causal pathways. Future studies should validate mental health instruments through cognitive interviewing with minority elders and employ geospatial analytics to quantify healthcare accessibility barriers.

## 5 Conclusion

This study reveals ethnic minority older adults (Jing, Zhuang, Yao, Mulao) in Guangxi, China, face 2 ~ 3 times higher anxiety/depression risks than Han Chinese, driven by structural inequities (geographic isolation, cultural-linguistic barriers) and socioeconomic deprivation. Key risk factors include female gender, chronic multimorbidity, cognitive dysfunction, poor psychological resilience and negative aging attitudes, while occasional exercise, child support and medical insurance emerged as vital protective buffers. We propose a culturally adaptive intervention framework integrating: (1) AI-powered multilingual screening platforms; (2) bilingual cultural coordinators bridging traditional/modern care; (3) digital-physical hybrid clinical networks; (4) policy reforms ensuring 85% insurance coverage for mental health services. Although cross-sectional data limit causal analysis, this first Social Determinants of Health (SDH)-based study in China’s ethnic aging context provides actionable strategies to address healthcare inequities. Future work should validate interventions through longitudinal designs and quantify historical trauma’s role. This model offers a blueprint for achieving health equity in multiethnic aging societies globally.

## Data Availability

The original contributions presented in the study are included in the article/supplementary material, further inquiries can be directed to the corresponding author.
